# Complex Case of Open Fracture-Dislocation of the Elbow

**DOI:** 10.1155/2019/3495742

**Published:** 2019-05-13

**Authors:** Tommy Primeau, Philippe Beauchamp-Chalifour, Stéphane Pelet

**Affiliations:** ^1^Centre de Recherche FRQS du CHU de Québec—Hôpital Enfant-Jésus, 1401, 18ème Rue, (Québec), Québec, Canada G1J 1Z4; ^2^Department of Orthopedic Surgery, CHU de Québec—Hôpital Enfant-Jésus, 1401, 18ème Rue, (Québec), Québec, Canada G1J 1Z4

## Abstract

Complex elbow instability is difficult to surgically address. Careful consideration of the fractures and soft tissue injuries is required. We present the case of a patient who sustained an open fracture-dislocation of the elbow with significant loss of the external humeral condyle and partial loss of the olecranon. He was surgically treated with an iliac crest tricortical autograft fixed with a buttress plate and a lag screw. His lateral ulnar collateral ligament was reconstructed with tendinous autograft collected from his third and fourth extensor *digitorum longus* tendons. While the procedure complicated with a *Nocardia* infection and wound breakdown, the patient almost had full range of motion without instability at 11 months of follow-up.

## 1. Introduction

The elbow is one of the most commonly dislocated joints, with an incidence of acute dislocation of 5.21 per 100 000 person-years [1]. Complex elbow instability consists of one or many osteoarticular fractures associated with capsular, ligament, and musculotendinous injuries, resulting in a global elbow instability [2].

The radial head and the coronoid process are the most commonly fractured structures in these injuries [3]. Six main types of complex elbow instability have been described: (1) radial head fractures with ligament injuries with or without dislocation, (2) coronoid process fractures with medial collateral ligament injury, (3) terrible triad injuries, (4) posterior Monteggia lesions, (5) transolecranon fracture-dislocation, and (6) shear fractures of the humerus with collateral ligament injury with or without dislocation [2].

Open fracture-dislocation of the elbow is still a very rare entity, and few case reports have described such injuries [4]. Thus, the optimal treatment for those patients remains unclear.

Management of these types of injury is complex and requires consideration of the fractures and soft tissue components. Improper management increases the risk of chronic elbow instability and posttraumatic arthritis [5].

This report presents a case of a patient with an open fracture-dislocation of the elbow with significant loss of the external humeral condyle and partial loss of the olecranon. To our knowledge, little information is available about the management of such case, and no similar cases have been previously described.

## 2. Case Report

**Figure 1:**
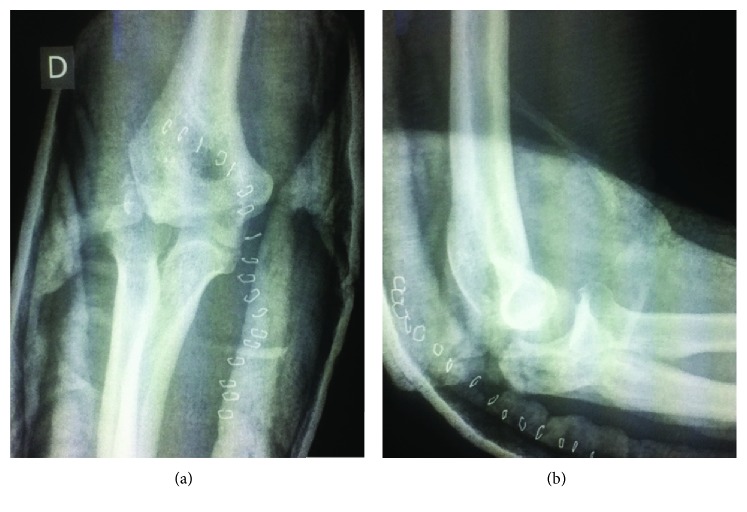
(a) Anteroposterior view of the elbow and (b) lateral view of the elbow following surgical debridement and partial olecranon excision.

A 39-year-old man was involved in a high-velocity motor vehicle accident. He sustained an open fracture of the right elbow, with significant loss of the external humeral condyle and partial loss of the olecranon. This fracture was classified as a *Gustillo* type IIIA injury. There was no neurovascular compromise. The patient was treated in a community center, close to the accident, where he received surgical care (debridement and partial excision of the olecranon). The wound was fully closed. The upper arm was then immobilized in a splint (Figure 1). IV antibiotics (cefazolin-gentamicin) were started for 5 days.

**Figure 2:**
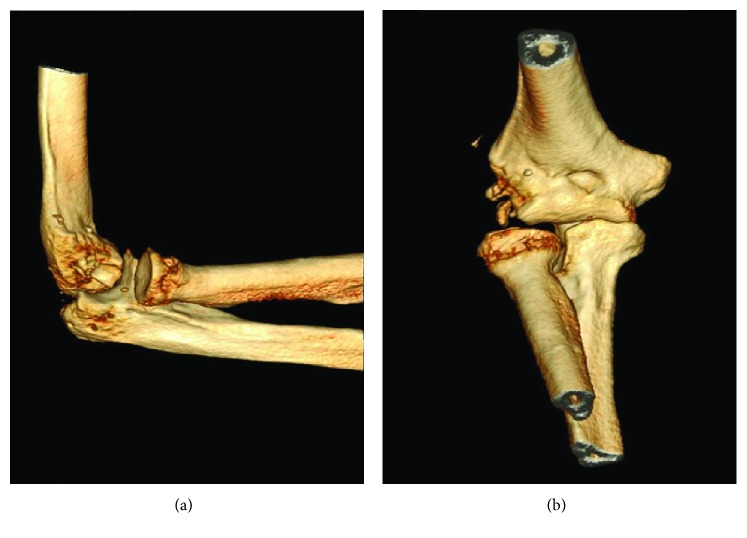
(a) Lateral view and (b) anterior view of preoperative *3-D CT scan* reconstruction showing external condyle loss and radial head subluxation.

The day following his elbow surgery, the patient fell in a staircase and sustained a C7-C8 and C8-T1 fracture-dislocation. This injury caused neurologic damage (quadriparesis), and his right arm became his only functional limb. Following this injury, the patient was moved to our tertiary center to get spinal fusion. During the spinal surgery, the elbow was tested under fluoroscopy. The patient's elbow showed varus instability and a positive pivot shift test. A CT scan of the elbow was obtained the following day and showed bony loss from the external humeral condyle and subluxation of the radial head (Figure 2).

**Figure 3:**
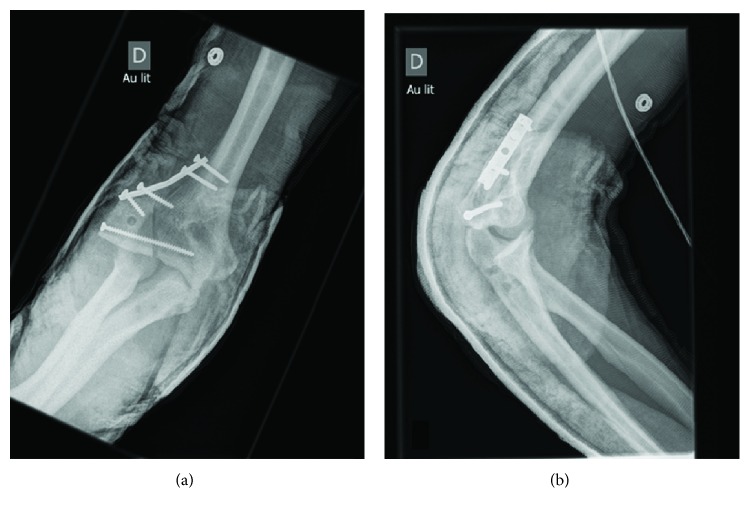
(a) Anteroposterior and (B) lateral 14 days postoperative X-rays of the right elbow.

We decided to treat the patient's elbow surgically. The surgery underwent nine days after the initial trauma (after transfer from the community center, spine procedure and elbow imaging). A posterior approach to the elbow was used along with an extensive elbow debridement. A tricortical iliac crest graft was then collected from the patient's right side to replace the humeral condyle bone loss. A tendinous graft was collected from his third and fourth extensor *digitorum longus* tendons to reconstruct the lateral collateral ligament. The tendinous graft was fixed to the bone graft through two tunnels (anterior to posterior and lateral to medial). The iliac crest graft was then fixed to the humerus with a cancellous screw. A five-hole one-third tubular plate was used as buttress. The graft was left prominent on the lateral side to improve lateral stability of the elbow. The lateral collateral ligament was then reconstructed by fixing the tendinous graft to the proximal ulna with two bundles. We took care to preserve our reconstructed lateral collateral ligament's isometry during flexion and extension movements. Reduction and stability of the elbow joint were verified under fluoroscopy. The wound was closed with staples, and a posterior splint was applied for three weeks. 14 days postoperative X-rays are presented on Figure 3. At three weeks following the surgery, passive and active ranges of motion were initiated.

While wearing the splint, the patient developed a pressure wound on the lateral side of his elbow (at the site of the reconstruction). This wound later complicated into a *Nocardia* septic arthritis, which required surgical debridement. Four months after the reconstruction, range of motion was 30-120 degrees with full pronation and supination, without any evidence of instability.

**Figure 4:**
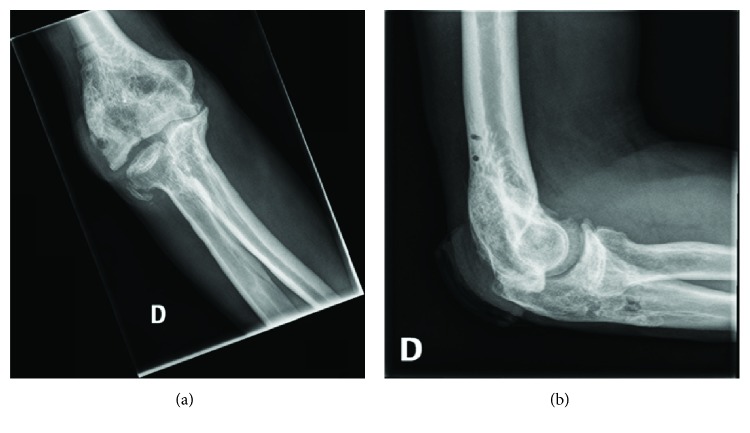
(a) Anteroposterior and (b) lateral 11 months postoperative X-rays of the right elbow.

Two months later, instrumentation was removed and a rotational flap was done to increase soft tissue coverage of the articulation. Five months after that procedure, the patient developed a *Nocardia* osteomyelitis of the humerus and was treated with 6 weeks of IV antibiotics (penicillin) and a surgical debridement. The patient responded well to our treatment. Eleven months after the initial intervention, range of motion of the joint ranged from 30 to 120 degrees of flexion, without clinical instability. Osteosynthesis was achieved according to X-rays of the elbow (Figure 4).

## 3. Discussion

Complex elbow instability is a very difficult pathology to surgically address. Morrey and An showed that the primary elbow stabilizers are the coronoid process, the olecranon, the humeral trochlea, and the collateral ligaments, while the secondary stabilizers are the radial head, the capitulum, the anterior capsule, the musculotendinous units, and the interosseous membrane [6]. Thus, it is essential to consider fractures as well as the soft tissue injuries in the treatment of complex elbow instability.

In our case, function restoration of the right elbow was especially important. The right arm was our patient's most useful limb considering his quadriparesis. As in every elbow injury, our main goals were to achieve stable osteosynthesis, correct and stable reduction, and to allow early range of motion.

Several options were possible for the bony reconstruction. Hattori et al. described a case of distal humeral fracture with significant bony loss treated with a tricortical iliac crest autograft [7]. They reported a good functional evolution at two years, despite the absence of articular cartilage [7]. Iliac crest autograft is also associated with a low risk of complications and allows surgeons to use a tricortical graft with a good amount of cancellous bone [8]. For those reasons, we choose this option for our bony reconstruction. As for other possible bone autografts, a case of bony reconstruction using a 1st metatarsophalangeal joint autograft for the elbow was described, but this technique resulted in important lateral instability following the surgery [9]. A total elbow arthroplasty or hemiarthroplasty is generally chosen in cases of low-demand elderly patients with osteoporosis [10, 11]. The high rate of complications associated with elbow arthroplasty, such as ulnar nerve injury, intraoperative fractures, triceps rupture, deep infection, and important functional limitations oriented our choice away from that option [10, 11]. As for arthrodesis, it is mainly considered a salvage procedure because of the complications and functional limitations associated [12]. In this case, since that arm needed to be the most functional one, we felt that arthrodesis was not the best solution. We also could have used allografts to reconstruct the bony defect, but the high rate of nonunion, resorption, and infection, especially in the case of an open fracture, led us away from that option [13].

O'Driscoll et al. have shown that the lateral ulnar collateral ligament is the main posterolateral stabilizer of the elbow, thus stressing the importance of its reconstruction [14]. Studies have shown that there is no advantage conferred by the use of a single or double-bundle reconstruction [15]. Classically, the *palmaris longus* is used, but was nonexistent in this patient [16]. The use of toe extensors has been described for collateral ligaments of the elbow but accounts for less than 2% of all autograft used according to a recent review of the literature and meta-analysis of 619 patients [17–19]. We although found the use of the third and fourth extensor *digitorum longus* tendons practical because of its easy retrieval and the fact that the patient would not be able to use his legs because of his spinal cord injury. *Gracilis* tendon, *semitendinous* tendon, triceps tendon, *plantaris* tendon, and extensor *carpi radialis longus* tendon have also been used for elbow ligament reconstruction [19]. However, we thought they would be harder to retrieve and add morbidity to the surgery. Allografts, especially the extensor *hallucis longus*, have also been used with good functional results [20]; although, they were used in chronic setting [20]. Synthetic grafts are still experimental options for elbow ligament reconstruction but have shown promising results [21]. Ten consecutive cases of chronic elbow instability have been surgically treated with a synthetic polyester ligament [21]. They were all stable at 27 months following surgery [21]. Many techniques have been described to reconstruct the lateral ulnar collateral ligament, but none was shown to be superior to the other (single versus double-stranded reconstruction) [15]. As for the tunnel to fix the ligament, its ideal location remains unclear due to prominence variability of the tubercule of the supinator crest [22]. A recent cadaveric study of seven patients comparing the stability of 70 tunnels using a double-stranded lateral ulnar collateral ligament found that reasonable elbow stability could be achieved as long as 1 of the 2 ulnar tunnels was located at or distal to the radial head-neck junction [23].

Following the surgery, the patient developed a pressure wound followed by infection because of the use of a splint. To avoid such complications, an elbow joint-overlapping external fixation as a first-staged fixation could have been used. Although a retrospective comparative study of 24 patients with an open or closed distal humerus fracture compared the use of primary external fixation and second-staged open reduction and internal fixation (ORIF) versus initial definitive ORIF [24]. With a median follow-up of 37 months, the study demonstrated higher complication rate and greater loss of extension for patients in the external fixation group (39 versus 17 degrees, *p* = 0.048, [24]. In our case, the reconstruction of the lateral epicondyle with an iliac crest autograft added to the complexity of the fracture, which drew us away from external fixation.

Complex elbow instability is a very challenging pathology for orthopaedic surgeons, especially in the case of an open fracture-dislocation. To our knowledge, no such case has been reported and makes the management of such injury difficult. We feel that the use of a tricortical iliac crest autograft to restore the bony architecture and the use of the third and fourth extensor *digitorum longus* tendons gave us the best chance to achieve good functional outcome for our patient. This was confirmed by the autograft osteointegration, the maintained reduction, the absence of instability, and the acceptable range of motion obtained at the eleven-month follow-up.

## References

[1] Stoneback J. W., Owens B. D., Sykes J., Athwal G. S., Pointer L., Wolf J. M. (2012). Incidence of elbow dislocations in the United States population. *The Journal of Bone and Joint Surgery-American Volume*.

[2] Giannicola G., Sacchetti F. M., Greco A., Cinotti G., Postacchini F. (2010). Management of complex elbow instability. *Musculoskeletal Surgery*.

[3] Hildebrand K. A., Patterson S. D., King G. J. W. (1999). Acute elbow dislocations : simple and complex. *Orthopedic Clinics of North America*.

[4] Shivanna D., Aski B., Manjunath D., Bhatnagar A. (2014). A rare combination open fracture dislocation of elbow with open fracture both bones forearm with radial nerve palsy. *Journal of Orthopaedic Case Reports*.

[5] Broberg M. A., Morrey B. F. (1987). Results of treatment of fracture-dislocations of the elbow. *Clinical Orthopaedics and Related Research*.

[6] Morrey B. F., an K. N. (1983). Articular and ligamentous contributions to the stability of the elbow joint. *The American Journal of Sports Medicine*.

[7] Hattori Y., Doi K., Pagsaligan J. M., Takka S., Ikeda K. (2005). Arthroplasty of the elbow joint using vascularized iliac bone graft for reconstruction of massive bone defect of the distal humerus. *Journal of Reconstructive Microsurgery*.

[8] Cavadas P. C., Landin L., Thione A., Ibañez J., Nthumba P., Roger I. (2010). Reconstruction of massive bone losses of the elbow with vascularized bone transfers. *Plastic and Reconstructive Surgery*.

[9] Shibata M. (2003). Elbow joint reconstruction using metatarsophalangeal joint of the great toe. *Experimental and Clinical Reconstructive Microsurgery*.

[10] Müller L. P., Kamineni S., Rommens P. M., Morrey B. F. (2005). Primary total elbow replacement for fractures of the distal humerus. *Operative Orthopädie und Traumatologie*.

[11] Burkhart K. J., Nijs S., Mattyasovszky S. G. (2011). Distal humerus hemiarthroplasty of the elbow for comminuted distal humeral fractures in the elderly patient. *The Journal of Trauma: Injury, Infection, and Critical Care*.

[12] Reichel L. M., Wiater B. P., Friedrich J., Hanel D. P. (2011). Arthrodesis of the elbow. *Hand Clinics*.

[13] Ehsan A., Lee B., Itamura J. M. (2010). Total elbow allografts with collateral ligament reconstruction for posttraumatic elbow injuries. *Journal of Orthopaedic Science*.

[14] O’Driscoll S. W., Bell D. F., Morrey B. F. (1991). Posterolateral rotatory instability of the elbow. *The Journal of Bone and Joint Surgery American Volume*.

[15] King G. J. W., Dunning C. E., Zarzour Z. D. S., Patterson S. D., Johnson J. A. (2002). Single-strand reconstruction of the lateral ulnar collateral ligament restores varus and posterolateral rotatory stability of the elbow. *Journal of Shoulder and Elbow Surgery*.

[16] Erickson B. J., Harris J. D., Chalmers P. N. (2015). Ulnar collateral ligament reconstruction: anatomy, indications, techniques, and outcomes. *Sports Health*.

[17] Azar F. M. (2001). Operative treatment of ulnar collateral ligament injuries of the elbow in athletes. *Operative Techniques in Orthopaedics*.

[18] Rohrbough J. T., Altchek D. W., Hyman J., Williams R. J., Botts J. D. (2002). Medial collateral ligament reconstruction of the elbow using the docking technique. *The American Journal of Sports Medicine*.

[19] Hagemeijer N. C., Claessen F. M. A. P., de Haan R., Riedijk R., Eygendaal D. E., van den Bekerom M. P. (2017). Graft site morbidity in elbow ligament reconstruction procedures: a systematic review. *The American Journal of Sports Medicine*.

[20] Conti Mica M., Caekebeke P., van Riet R. (2016). Lateral collateral ligament injuries of the elbow—chronic posterolateral rotatory instability (PLRI). *EFORT Open Reviews*.

[21] Tawari G. J. K., Lawrence T., Stanley D. (2013). Surgical reconstructions for posterolateral rotatory instability of elbow using a synthetic ligament. *Shoulder & Elbow*.

[22] Anakwenze O. A., Khanna K., Levine W. N., Ahmad C. S. (2014). Characterization of the supinator tubercle for lateral ulnar collateral ligament reconstruction. *Orthopaedic Journal of Sports Medicine*.

[23] Kim H. M., Andrews C. R., Roush E. P., Pace G. I., Lewis G. S. (2017). Effect of ulnar tunnel location on elbow stability in double-strand lateral collateral ligament reconstruction. *Journal of Shoulder and Elbow Surgery*.

[24] Kösters C., Lenschow S., Schulte-Zurhausen E., Roßlenbroich S., Raschke M. J., Schliemann B. (2017). Management of comminuted fractures of the distal humerus: clinical outcome after primary external fixation versus immediate fixation with locking plates. *Archives of Orthopaedic and Trauma Surgery*.

